# Pharmacist-led management of suspected drug-provoked seizures and sequential antiseizure medication adverse reactions: a case report

**DOI:** 10.3389/fphar.2026.1853932

**Published:** 2026-06-29

**Authors:** Mimi Wang, You Wu, Xilin Xin, Zhangxuan Shou, Jueyi Pan

**Affiliations:** 1 Department of Pharmacy, The Second Affiliated Hospital of Zhejiang Chinese Medical University, Hangzhou, China; 2 Department of Neurology, The Second Affiliated Hospital of Zhejiang Chinese Medical University, Hangzhou, China

**Keywords:** clinical pharmacist, diprophylline, drug-provoked seizure, pharmacovigilance, renal impairment

## Abstract

**Background:**

Drug-provoked seizures constitute a clinically significant yet frequently underappreciated complication of pharmacotherapy, particularly among patients receiving antimicrobial agents with established seizurogenic potential. The subsequent management of sequential adverse drug reactions (ADRs) arising from antiseizure medication (ASM) use introduces additional pharmacological and clinical complexity.

**Case Summary:**

A 73-year-old man with a remote history of epilepsy, moderate chronic kidney disease (CKD), and decade-long anticonvulsant self-discontinuation experienced two generalised tonic-clonic seizures during sleep on completion of a five-day course of intravenous moxifloxacin 0.4 g once daily and intravenous diprophylline 0.5 g twice daily for an acute COPD exacerbation. The clinical pharmacist identified the events as consistent with suspected drug-provoked seizures, hypothesising that renal impairment-driven diprophylline accumulation may have acted as a pharmacokinetically amplified pro-convulsant mechanism; as serum diprophylline concentrations were not measured, this mechanism is proposed as hypothesis-generating rather than established. Three sequential ASM-associated ADRs were subsequently detected and managed through proactive pharmacist-led pharmacovigilance: suspected sodium valproate-induced rhabdomyolysis (Naranjo score 8; confounded by post-ictal rhabdomyolysis and concurrent statin use), symptomatic oxcarbazepine-induced hyponatraemia requiring drug substitution, and suspected levetiracetam-associated hepatotoxicity not fulfilling formal drug-induced liver injury (DILI) criteria.

**Conclusion:**

This case underscores the indispensable role of clinical pharmacist engagement in identifying suspected drug-provoked seizures, applying structured causality assessment in the presence of competing confounders, and systematically managing complex, sequential ASM-related adverse reactions in high-risk patients.

## Introduction

Drug-provoked seizures—epileptic events precipitated by an identifiable exogenous agent—represent a clinically important yet frequently underappreciated complication of pharmacotherapy. Unlike unprovoked epileptic seizures, drug-provoked events arise from a time-limited pharmacological insult, and their timely identification carries direct implications for drug selection, dose adjustment, and recurrence prevention.

Fluoroquinolones and methylxanthine bronchodilators are among the drug classes most frequently implicated in lowering seizure thresholds, and are commonly co-administered in the management of acute COPD exacerbations. Moxifloxacin, a fourth-generation fluoroquinolone widely used for respiratory tract infections, has been reported as a precipitant of *de novo* seizures in neurologically vulnerable patients (Marques et al., 2025). Diprophylline—in contrast to theophylline—is excreted predominantly as unchanged drug by the kidneys and undergoes minimal hepatic biotransformation; its systemic exposure is therefore directly proportional to residual renal function, a pharmacokinetic characteristic of particular clinical relevance in patients with chronic kidney disease (CKD). When ASM therapy is initiated following a drug-provoked seizure, clinical complexity deepens further: each agent carries a distinct adverse-effect profile, and sequential ADRs—including rhabdomyolysis, electrolyte disorders, and hepatotoxicity—are individually well-documented but rarely reported as a continuous, pharmacist-detected cascade in a single patient.

We herein report a case in which a clinical pharmacist identified suspected drug-provoked seizures in a 73-year-old man with COPD and moderate CKD receiving concurrent intravenous moxifloxacin and diprophylline, against a background of decade-long anticonvulsant self-discontinuation. Through case analysis, we propose a previously unreported pharmacokinetic amplification mechanism (hypothesis-generating, as serum diprophylline concentrations were not measured), detail the sequential pharmacist-led management of three mechanistically distinct ASM-associated ADRs, and demonstrate the indispensable contribution of the clinical pharmacist within the multidisciplinary team. This report adheres to the CARE Case Report guidelines; a completed CARE checklist is submitted as supplementary material.

## Case presentation

### Patient information and clinical findings

A 73-year-old man (height 165 cm, weight 51 kg; body mass index 18.7 kg/m^2^; fully de-identified) was admitted to the Neurology Department on 16 December 2024 following two witnessed generalised tonic-clonic seizure episodes during sleep the preceding night. The principal presenting complaint comprised recurrent loss of consciousness accompanied by bilateral tonic-clonic limb movements. He had received a diagnosis of epilepsy 3 decades earlier and was initially managed with sodium valproate sustained-release tablets 125 mg once daily, which he had self-discontinued more than 10 years prior to the current admission.

Relevant medical history comprised hypertension (currently untreated), anxiety disorder and sleep disturbance (estazolam 1 mg nightly; trazodone 16.7 mg nightly), and carotid atherosclerosis with plaque formation (pitavastatin calcium 2 mg once daily). His social history was notable for a more than 40-year history of smoking 15 cigarettes daily (approximately 30 pack-years), having ceased 5 years before admission, with no alcohol use. Between 10 and 15 December 2024, the patient had been hospitalised in the Respiratory Medicine Department for an acute COPD exacerbation and received moxifloxacin sodium chloride injection 0.4 g once daily (intravenous drip) and diprophylline injection 0.5 g twice daily (intravenous drip) for five consecutive days.

On Neurology admission at approximately 04:00 on 16 December, vital signs were as follows: temperature 37.2 °C, heart rate 89 beats/min, respiratory rate 19 breaths/min, and blood pressure 160/95 mmHg. Neurological examination revealed no focal deficits. Key laboratory values on admission included serum creatinine 136.6 μmol/L (estimated glomerular filtration rate [eGFR] 32.09 mL/min/1.73 m^2^ by the CKD-EPI equation, consistent with CKD stage 3b), potassium 3.84 mmol/L, sodium 138.10 mmol/L, and AST 23 U/L. Baseline investigations from the preceding respiratory admission on 10 December are presented in Table 1; notable values included elevated serum creatinine (143.5 μmol/L) and amylase (153 U/L), with a normal creatine kinase (CK; 63 U/L). Non-contrast CT of the brain performed at 03:55 on 16 December demonstrated bilateral periventricular ischaemic foci, a possible left-sided lacunar infarct, and age-related cerebral changes. Brain MRI on 17 December revealed multifocal white matter hyperintensities (WMH) in the brainstem, bilateral periventricular regions, and centrum semiovale (Fazekas grade 3), consistent with severe leukoaraiosis. No electroencephalogram (EEG) was performed during this admission; both episodes had self-terminated before transfer to the Neurology Department, the patient remained haemodynamically stable and seizure-free thereafter, and the immediate clinical priority was the identification and withdrawal of the suspected precipitating drugs; in addition, as no further episodes occurred, the patient declined EEG examination. The seizure classification therefore rests on the witnessed clinical semiology and was not electrographically confirmed. Admission diagnoses included status epilepticus, bilateral carotid atherosclerosis with plaques, hypertension, COPD, anxiety disorder, and sleep disturbance.

**TABLE 1:** Medication timeline, clinical events, and key laboratory values during hospitalisation.

Date	Medication changes	Symptoms/Signs	Key laboratory values	Pharmacist actions
10 December 2024 (Resp. Dept.)	Moxifloxacin 0.4 g once daily IV drip; diprophylline 0.5 g twice daily IV drip (10–15 December)	Acute COPD exacerbation	K^+^ 4.57 mmol/L; Na^+^ 137.20 mmol/L; Cl^−^ 103.60 mmol/L; Cr 143.5 μmol/L↑; ALT 13 U/L; AST 21 U/L; CK 63 U/L; CK-MB 9.5 U/L; LDH 174 U/L; amylase 153 U/L↑; RBC 4.15 × 10^12^/L; Hb 134 g/L	—
15 December 22:00	Final day of moxifloxacin + diprophylline (both IV)	Episode 1: Generalised tonic-clonic seizure during sleep; upward gaze deviation, bilateral limb jerking; duration ∼20 min; self-terminated	No specimens obtained at time of seizure	—
16 December 03:50–04:00	Sodium valproate injection 400 mg IV pump (4 mL/h) commenced; sodium valproate SR 0.5 g twice daily oral added; transferred to neurology	Episode 2: Tonic-clonic seizure ∼5 min, self-terminated; transferred to neurology ∼04:00	K^+^ 3.84 mmol/L; Na^+^ 138.10 mmol/L; Cl^−^ 106.80 mmol/L; Cr 136.6 μmol/L↑; eGFR 32.09 mL/min/1.73 m^2^; AST 23 U/L; CK 68 U/L; CK-MB 18.1 U/L; LDH 161 U/L; Hb 122 g/L	Reviewed complete medication list; identified concurrent pro-convulsant agents (moxifloxacin + diprophylline) and patient risk profile (CKD, prior epilepsy, polypharmacy); communicated findings to medical team
16 December 03:55 (CT) 17 December 10:00 (MRI)	No new medications	CT: Bilateral periventricular ischaemic foci; possible left lacunar infarct; age-related changes. MRI: Multifocal WMH in brainstem, bilateral periventricular regions and centrum semiovale; Fazekas grade 3 (severe leukoaraiosis). No EEG performed (declined by patient; no further episodes)	K^+^ 4.93 mmol/L; Na^+^ 137.2 mmol/L; Cl^−^ 102.9 mmol/L; Cr 144.8 μmol/L; ALT 32 U/L; AST 26 U/L; CK 50 U/L; CK-MB 12.8 U/L; LDH 152 U/L; amylase 226 U/L; Hb 145 g/L	Documented absence of EEG; noted neuroimaging findings (Fazekas 3) as relevant to seizure threshold reduction; flagged CKD as pharmacokinetic risk modifier for potential diprophylline accumulation
18 December IV stopped 10:00 oral stopped 17:00	Valproate IV pump STOPPED at 10:00; last oral SR tablet taken AM; valproate SR STOPPED at 17:00; pitavastatin STOPPED; IV hydration + K^+^ supplementation initiated	Fever, chills, head fullness	CK 17,173 U/L↑↑↑; LDH 1,037 U/L↑; AST 225 U/L↑; ALT 41 U/L↑; NT-proBNP 1,550 pg/mL; valproate TDM 156 μg/mL [ref: 50–100 μg/mL]	TDM performed at 17:00 (∼9 h post last oral dose; ∼7 h post IV cessation): valproate 156 μg/mL [supratherapeutic]; naranjo scale applied: score 8 (‘probable’ ADR); identified two independent confounders: post-ictal rhabdomyolysis and statin myopathy; recommended immediate drug cessation and IV hydration
19 Dec	Oxcarbazepine 0.3 g twice daily commenced	Afebrile; no further seizures	CK 12,928 U/L↓; AST 191 U/L; LDH 635 U/L; K^+^ 3.48 mmol/L; Na^+^ 141.5 mmol/L	Recommended serial monitoring of serum electrolytes, liver function tests (LFTs), and complete blood count (CBC); flagged elevated age-related risk of oxcarbazepine-induced hyponatraemia
28–29 Dec	KCl supplementation (IV + oral) added; oxcarbazepine reduced to 0.15 g twice daily (29 December)	Limb weakness; falls; chest tightness; exertional dyspnoea	K^+^ 2.42→2.87 mmol/L↓; Na^+^ 135.5→127.2 mmol/L↓; Cl^−^ 92.6→90.1 mmol/L↓; plasma osmolality 271.3 mOsm/kg	Identified symptomatic oxcarbazepine-induced hyponatraemia and concomitant hypokalaemia; recommended immediate oxcarbazepine dose reduction and aggressive electrolyte supplementation
30 Dec	Oxcarbazepine DISCONTINUED; levetiracetam 0.5 g twice daily commenced; oral 10% NaCl supplementation added	Clinical improvement; persistent mild dyspnoea	K^+^ 3.45 mmol/L; Na^+^ 133.1 mmol/L	Recommended levetiracetam as replacement ASM on basis of renal elimination profile, absence of CYP450 induction, and minimal drug interaction potential; proposed continued electrolyte monitoring with planned NaCl taper
1 January 2025	Levetiracetam reduced to 0.25 g twice daily; polyene phosphatidylcholine 228 mg three times daily added	Electrolytes normalised; mild transaminase elevation detected	ALT 99 U/L↑; AST 92 U/L↑; CK 39 U/L; K^+^ 4.06 mmol/L; Na^+^ 140.7 mmol/L	Identified elevated transaminases 2 days post-levetiracetam initiation; formal DILI diagnostic criteria not fulfilled; recommended dose reduction and hepatoprotective therapy (polyene phosphatidylcholine, per 2023 Chinese DILI guidelines) in preference to glycyrrhizinate preparations given prior electrolyte instability
3 Jan (discharge)	Levetiracetam 0.25 g twice daily; polyene phosphatidylcholine 228 mg three times daily; trazodone 25 mg nightly	No seizures; fatigue improving	—	Discharge medication counselling provided; outpatient review arranged 14 January 2025; seizure diary initiated
14 January (OPD)	Continued discharge regimen unchanged	No seizure recurrence; subjective improvement in energy	K^+^ 4.93 mmol/L; Na^+^ 137.2 mmol/L; Cl^−^ 102.9 mmol/L; Cr 144.8 μmol/L; ALT 32 U/L (normalised); AST 26 U/L (normalised); CK 50 U/L (normalised); CK-MB 12.8 U/L; LDH 152 U/L (normalised); Hb 145 g/L	Confirmed complete hepatic and metabolic biochemical recovery at 11-day post-discharge review; continued outpatient seizure surveillance planned

Abbreviations: ADR, adverse drug reaction; ASM, antiseizure medication; bid, twice daily; CBC, complete blood count; CK, creatine kinase (U/L); CK-MB, creatine kinase-MB, isoenzyme (U/L); Cl^−^, chloride (mmol/L); CKD, chronic kidney disease; COPD, chronic obstructive pulmonary disease; Cr, creatinine (μmol/L); DILI, drug-induced liver injury; EEG, electroencephalogram; eGFR, estimated glomerular filtration rate (mL/min/1.73 m^2^); Hb, haemoglobin (g/L); IV, intravenous; K^+^, potassium (mmol/L); LDH, lactate dehydrogenase (U/L); LFTs, liver function tests; Na^+^, sodium (mmol/L); NT-proBNP, N-terminal pro-B-type natriuretic peptide (pg/mL); OPD, outpatient department; RBC, red blood cell count (×10^12^/L); SR, sustained-release; TDM, therapeutic drug monitoring; WMH, white matter hyperintensities. Reference ranges: ALT <40 U/L; AST <40 U/L; CK, 26–200 U/L; LDH, 120–250 U/L; K^+^ 3.5–5.5 mmol/L; Na^+^ 135–145 mmol/L; Cr (male) 62–115 μmol/L ↑ above reference range; ↓ below reference range; ↑↑↑ markedly elevated.

### Timeline and therapeutic interventions

**FIGURE 1:**
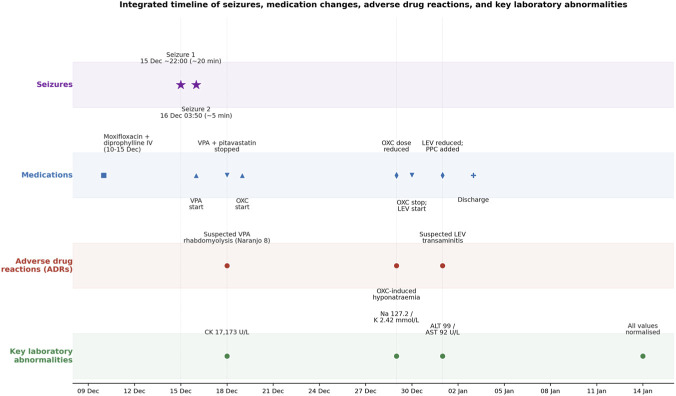
Integrated graphical timeline of the hospital course, consolidating the four event streams otherwise tabulated in Table 1. The horizontal axis spans the respiratory admission (10 December 2024) to the post-discharge outpatient review (14 January 2025). The lanes display, from top to bottom: the two witnessed seizure episodes; key medication changes (initiation, dose reduction, discontinuation, and substitution of sodium valproate, oxcarbazepine, and levetiracetam, and withdrawal of pitavastatin); the three sequential antiseizure-medication-associated adverse drug reactions (suspected valproate-associated rhabdomyolysis, oxcarbazepine-induced hyponatraemia, and suspected levetiracetam-associated transaminase elevation); and the corresponding peak laboratory abnormalities (creatine kinase 17,173 U/L; sodium nadir 127.2 mmol/L; potassium nadir 2.42 mmol/L; alanine aminotransferase 99 U/L), all of which had normalised by the 14 January review. ALT, alanine aminotransferase; AST, aspartate aminotransferase; CK, creatine kinase; K, potassium; LEV, levetiracetam; Na, sodium; OXC, oxcarbazepine; PPC, polyene phosphatidylcholine; VPA, sodium valproate.

The complete medication timeline and serial laboratory results are presented in Table 1 and are additionally summarised in the integrated graphical timeline (Figure 1). The first seizure episode occurred on 15 December 2024 at approximately 22:00 during sleep: generalised tonic-clonic convulsions with upward gaze deviation and bilateral limb jerking lasting approximately 20 min, self-terminating without pharmacological intervention. A second episode of similar semiology occurred on 16 December at 03:50, lasting approximately 5 min, also resolving spontaneously.

Following transfer to the Neurology Department on 16 December, sodium valproate injection 400 mg was initiated as a continuous intravenous pump infusion (4 mL/h), in conjunction with sodium valproate sustained-release tablets 0.5 g twice daily orally. On 18 December, serum CK rose markedly to 17,173 U/L. Valproate TDM was performed on 18 December at 17:00, approximately 9 hours after the last oral dose and 7 hours after cessation of the intravenous infusion at 10:00; the measured serum concentration of 156 μg/mL substantially exceeded the established therapeutic reference range for epilepsy (50–100 μg/mL; immunoassay). Causality was formally assessed using the Naranjo Adverse Drug Reaction Probability Scale, yielding a score of 8 (“probable”). On 18 December, all valproate formulations and pitavastatin were discontinued, and intravenous hydration was initiated.

Oxcarbazepine 0.3 g twice daily was commenced on 19 December. By 28–29 December, the patient developed symptomatic hyponatraemia (nadir sodium 127.2 mmol/L) and hypokalaemia (nadir potassium 2.42 mmol/L), with associated limb weakness, falls, and dyspnoea. Oxcarbazepine was reduced to 0.15 g twice daily on 29 December, with addition of oral and intravenous potassium chloride supplementation. On 30 December, oxcarbazepine was discontinued and replaced with levetiracetam 0.5 g twice daily, with oral 10% sodium chloride supplementation initiated.

On 1 January 2025, 2 days after levetiracetam initiation, mild transaminase elevation was detected (ALT 99 U/L; AST 92 U/L). Levetiracetam was reduced to 0.25 g twice daily, and polyene phosphatidylcholine 228 mg three times daily was added as hepatoprotective therapy. The patient was discharged on 3 January 2025 on levetiracetam 0.25 g twice daily, polyene phosphatidylcholine 228 mg three times daily, and trazodone 25 mg nightly.

### Diagnostic assessment

**FIGURE 2:**
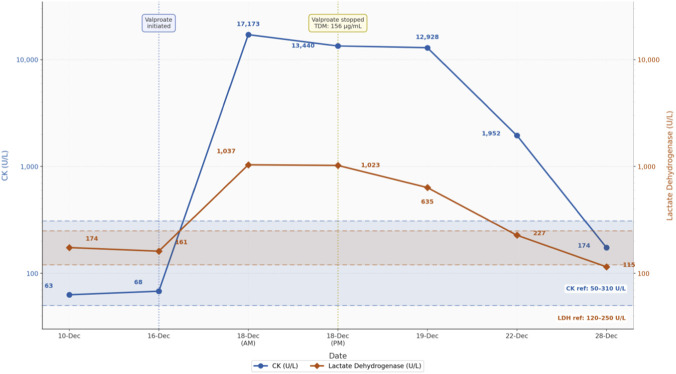
Serial trends in serum creatine kinase (CK; U/L) and lactate dehydrogenase (LDH; U/L) during hospitalisation. Horizontal dashed lines indicate upper reference limits (CK: 200 U/L; LDH: 250 U/L). Annotated events include sodium valproate initiation (16 December 2024), sodium valproate discontinuation and pitavastatin withdrawal (18 December 2024), and the serum valproate TDM result (156 μg/mL obtained at 17:00 on 18 December 2024, representing a supratherapeutic concentration 1.56-fold above the upper therapeutic limit of 100 μg/mL). CK, creatine kinase; LDH, lactate dehydrogenase; TDM, therapeutic drug monitoring.

**FIGURE 3:**
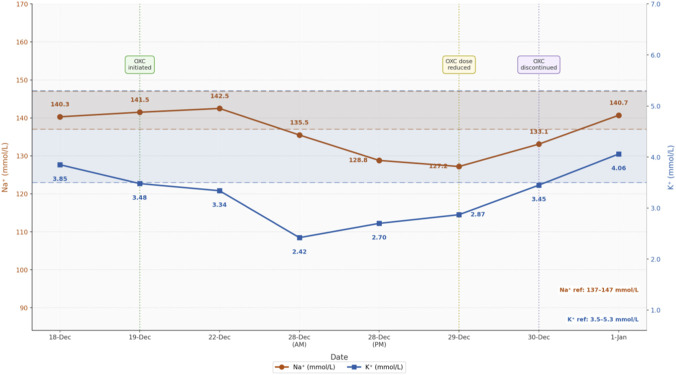
Serial trends in serum sodium (Na^+^; mmol/L) and potassium (K^+^; mmol/L) during hospitalisation. Horizontal dashed lines indicate lower reference limits (Na^+^: 135 mmol/L; K^+^: 3.5 mmol/L). Annotated events include oxcarbazepine initiation (19 December 2024), dose reduction to 0.15 g twice daily (29 December 2024), and discontinuation with substitution by levetiracetam (30 December 2024). K^+^, potassium; Na^+^, sodium.

Serial trends in CK, lactate dehydrogenase (LDH), and serum electrolytes are illustrated in Figures 2, 3, respectively. The differential diagnosis for the seizure episodes encompassed: (Čiauškaiteė et al., 2022): recurrence of primary epilepsy following decade-long anticonvulsant self-discontinuation—the single most important predisposing factor; (Deng et al., 2023); suspected drug-provoked seizure attributable to moxifloxacin and/or diprophylline, with hypothesised renal impairment-driven diprophylline accumulation as a pharmacokinetic modifier; (Gueta et al., 2024); metabolic disturbance—excluded, as serum electrolytes were within normal limits at the time of the episodes (sodium 138.10 mmol/L; potassium 3.84 mmol/L); (Hegazy et al., 2023); acute cerebrovascular event—considered unlikely given the absence of focal neurological deficits, though the incidentally identified possible left lacunar infarct was noted; and (Kim et al., 2014) CNS infection—excluded by the absence of fever, meningismus, and altered consciousness.

### Follow-up and outcomes

At outpatient review on 14 January 2025, eleven days after discharge, no further seizures had occurred. Laboratory investigations demonstrated complete biochemical recovery: ALT 32 U/L (from a peak of 99 U/L on 1 January), AST 26 U/L (from 92 U/L), CK 50 U/L (from a peak of 17,173 U/L), LDH 152 U/L, sodium 137.2 mmol/L, potassium 4.93 mmol/L, and serum creatinine 144.8 μmol/L. The patient reported progressive improvement in energy levels and the complete absence of neurological symptoms. All discharge medications were well tolerated. As this initial review covered only a short post-discharge interval, the patient has subsequently continued regular outpatient follow-up at our hospital, with no recurrence of seizures and sustained normalisation of the relevant biochemical investigations; longer-term surveillance of seizure control, hepatic and renal function, serum electrolytes, and quality of life and psychosocial functioning is ongoing.

## Discussion

This case exemplifies the diagnostic and therapeutic complexity that emerges when multiple pharmacological, neurological, and systemic seizure precipitants converge in a single high-risk patient. The decade-long self-discontinuation of anticonvulsant therapy represented the single most important predisposing determinant of seizure recurrence. Against this background of compromised seizure threshold, the patient received two pharmacological agents with well-characterised but mechanistically distinct pro-convulsant properties. Moxifloxacin exerts its seizurogenic effects through competitive inhibition of γ-aminobutyric acid type A (GABA-A) receptor binding and concurrent agonistic activity at N-methyl-D-aspartate (NMDA) receptors, thereby reducing the CNS excitability threshold (Wanleenuwat et al., 2020). Diprophylline lowers the seizure threshold through an independent pathway: antagonism of adenosine A1 receptors, which under physiological conditions provide tonic inhibitory modulation of neuronal excitability (Wanleenuwat et al., 2020). When co-administered, these two mechanisms act through pharmacodynamically additive but mechanistically distinct pathways, collectively depressing seizure threshold beyond what either agent alone would be expected to produce. Concurrent acute COPD exacerbation with systemic infection, hypoxaemia, sleep-state seizure onset, and neuroimaging evidence of severe leukoaraiosis (Fazekas grade 3) — a condition associated with substantially reduced cortical inhibitory reserve—likely contributed as non-pharmacological precipitants. It must be emphasised that these competing factors—remote epilepsy with decade-long anticonvulsant withdrawal, severe leukoaraiosis (Fazekas grade 3), an acute respiratory infection with probable hypoxaemia, advanced age, and polypharmacy—are each independently capable of provoking seizures in the absence of any pro-convulsant drug exposure, and several were probably operative concurrently. The pharmacological hypothesis advanced below therefore represents one of several plausible contributors rather than an established sole cause, and these alternative precipitants cannot be disentangled from the drug exposure on the basis of the available clinical data. Given this multifactorial constellation, definitive causal attribution to any single agent is not possible.

A clinically relevant mechanistic hypothesis generated by this case is the potential pharmacokinetic amplification of diprophylline’s pro-convulsant potential by concurrent renal impairment. In contrast to theophylline, which undergoes extensive CYP1A2-mediated hepatic biotransformation, diprophylline is eliminated predominantly as unchanged drug via renal excretion, with approximately 84%–88% of the administered dose recovered intact in the urine. In the setting of moderate renal impairment—as present in this patient (eGFR 32.09 mL/min/1.73 m^2^; CKD stage 3b) — reduced renal clearance would be expected to promote drug accumulation, potentially raising plasma concentrations above those anticipated under normal renal function and amplifying adenosine A1 receptor antagonism. However, because serum diprophylline concentrations were not measured in this patient, such accumulation is inferred from established pharmacokinetic principles rather than directly demonstrated. Critically, moxifloxacin neither inhibits CYP1A2 nor exerts any pharmacokinetic effect on methylxanthine plasma concentrations (Stass and Kubitza, 2001); the combined pro-convulsant risk of their co-administration is therefore exclusively pharmacodynamic in nature. This proposed pharmacokinetic–pharmacodynamic amplification mechanism, to our knowledge not previously described in the clinical context of concurrent fluoroquinolone and methylxanthine administration in a patient with CKD, should be regarded as hypothesis-generating rather than established. Irrespective of the precise mechanism, a prudent clinical implication follows: in patients with CKD receiving diprophylline, renal function-guided dose adjustment or substitution with an alternative bronchodilator that does not share this renal elimination dependency should be proactively considered.

The epidemiological evidence contextualises fluoroquinolone-associated seizure risk in a more nuanced framework. Large real-world cohort studies encompassing millions of patients have not demonstrated a statistically significant increase in overall seizure incidence with fluoroquinolones compared with amoxicillin in unselected populations (Tassopoulou et al., 2026; Gueta et al., 2024). However, pharmacovigilance signal analyses of the FDA Adverse Event Reporting System (FAERS) consistently confirm that CNS adverse events—seizures included—are disproportionately concentrated in patients sharing the profile present in this case: advanced age, renal impairment, pre-existing neurological disease, and polypharmacy (Xie et al., 2024). Collectively, these data establish that population-level safety estimates are not uniformly applicable to pharmacologically vulnerable subgroups, in whom individual risk-benefit evaluation is imperative. In navigating causality assessment within such complex, multi-precipitant clinical scenarios, the Naranjo Adverse Drug Reaction Probability Scale provides structured probabilistic attribution, while TDM supplies objective pharmacokinetic corroboration. The absence of EEG data is acknowledged as a methodological constraint that precluded formal electrographic seizure characterisation; nonetheless, the bilateral generalised semiology, the temporal alignment with pharmacological exposure, and the convergence of multiple established risk factors collectively provided sufficient grounds to classify these events as suspected drug-provoked seizures within a pharmacovigilance framework; the seizure type itself, however, remains a clinical rather than an electrographically confirmed classification.

The marked CK elevation—peaking at 17,173 U/L, representing an approximately 86-fold elevation above the upper reference limit—necessitated structured assessment for sodium valproate-associated rhabdomyolysis. Pharmacovigilance data from real-world registries confirm rhabdomyolysis as a documented, if uncommon, class-level adverse effect within the valproate family (Deng et al., 2023), corroborated by published case reports (Sharma et al., 2023; Walker and Deb, 2021; Shen et al., 2023). Formal causality assessment using the Naranjo scale yielded a score of 8 (‘probable’), reflecting a clear temporal association and a positive dechallenge response. However, two independent confounders must be explicitly acknowledged: post-ictal rhabdomyolysis—a well-recognised sequela of prolonged generalised tonic-clonic seizures—and pitavastatin-associated statin myopathy, each independently capable of producing severe CK elevation. Of particular note, the marked CK rise followed closely upon prolonged generalised tonic-clonic seizure activity, so post-ictal rhabdomyolysis is at least as plausible a primary explanation as valproate. The Naranjo score should therefore be interpreted with caution, as the scale does not adequately weight co-occurring non-pharmacological causes and may overestimate drug attributability when confounders overlap temporally with the suspected agent. The valproate TDM result of 156 μg/mL—obtained 9 hours after the last oral dose and 7 hours following intravenous cessation, representing neither a formal pharmacokinetic trough nor peak—substantially exceeded the established therapeutic range of 50–100 μg/mL. Although supratherapeutic valproate exposure is biologically plausible as a contributory mechanism, its relative clinical weight against two competing explanations cannot be definitively resolved from the available clinical data. Supplementary application of the WHO-UMC causality criteria or the RUCAM scale would afford more granular drug-attributability evaluation in such complex, multi-confounder scenarios.

Oxcarbazepine-induced hyponatraemia is the most prevalent ADR associated with this agent, with a reported incidence of 17.9%–73.3% across treated cohorts. The underlying mechanism involves enhanced vasopressin sensitivity in renal collecting tubule epithelium, culminating in inappropriate water retention (Čiauškaiteė et al., 2022). A large prospective cohort study encompassing 1,009 patients confirmed that advancing age is an independent predictor of both severe hyponatraemia (OR 1.014 per year of age; p = 0.014) and symptomatic hyponatraemia (OR 1.034 per year; p = 0.001) (Kim et al., 2014), placing this 73-year-old patient at substantially elevated baseline risk prior to drug initiation. These quantitative risk data reinforce the imperative for pre-emptive baseline electrolyte measurement and serial monitoring when oxcarbazepine is introduced in elderly patients. Furthermore, they support a low threshold for dose reduction or drug substitution when hyponatraemia persists or progresses despite active electrolyte supplementation—the approach implemented in this case.

Levetiracetam was selected as the replacement ASM on the basis of its renal elimination profile, independence from hepatic CYP450 enzyme induction or inhibition, and minimal drug–drug interaction liability—pharmacological attributes of particular value in a patient with established renal impairment and electrolyte fragility (Sun et al., 2022). The mild transaminase elevation observed 2 days after levetiracetam initiation did not meet established DILI diagnostic thresholds and was classified as suspected levetiracetam-associated hepatic biochemical abnormality, with residual hepatotoxicity from prior valproate exposure and ongoing systemic illness acknowledged as additional contributory factors. Moreover, the 2-day latency lies at the very earliest extreme of the reported range for idiosyncratic levetiracetam hepatotoxicity, which further reduces the likelihood of a direct levetiracetam effect and favours residual valproate hepatotoxicity together with the systemic inflammatory state as the more probable explanations for this transient, self-limiting biochemical abnormality. The most comprehensive systematic review of levetiracetam-associated DILI to date documents a severity spectrum extending from subclinical enzyme elevation to fulminant hepatic failure, with a proposed idiosyncratic, immune-mediated pathogenesis and a latency period spanning days to several months (Rogalewski et al., 2021); a subsequent case report further characterised this entity as drug-induced autoimmune hepatitis (Hegazy et al., 2023). Prompt dose reduction combined with initiation of polyene phosphatidylcholine—the hepatoprotective agent recommended by the 2023 Chinese Clinical Guidelines for Drug-Induced Liver Injury for hepatocellular-pattern DILI—achieved complete biochemical normalisation within 2 weeks. Polyene phosphatidylcholine was selected in preference to glycyrrhizinate-based preparations given the patient’s prior oxcarbazepine-related electrolyte instability and the recognised sodium-retaining properties of the latter drug class.

The sequential identification and management of three mechanistically distinct ASM-associated ADRs within a single patient and a compressed clinical timeframe underscores the indispensable value of continuous, proactive bedside pharmacovigilance. The clinical pharmacist’s contributions spanned the full therapeutic trajectory: identification of the suspected drug-provoked seizure aetiology and a proposed pharmacokinetic mechanistic basis; structured causality assessment at each ADR stage, incorporating and weighing all concurrent clinical confounders; TDM-guided valproate dosing decisions; evidence-based selection of a hepatologically safe hepatoprotective regimen; and prospective monitoring of pharmacokinetic and pharmacodynamic drug-interaction risks throughout the admission. This pharmacist-integrated multidisciplinary approach ensured clinical coherence from initial seizure recognition through to complete biochemical and symptomatic recovery, confirmed at the 11-day post-discharge outpatient review.

## Strengths and limitations

Strengths of this report include temporally precise documentation of a sequential ADR cascade with structured causality assessment at each stage; proposal of a novel pharmacokinetic–pharmacodynamic mechanistic hypothesis—renal impairment-amplified diprophylline pro-convulsant toxicity—not previously described in this clinical context; adherence to CARE reporting guidelines; and integration of the most recent 2024–2025 evidence base. Principal limitations are as follows: (Čiauškaiteė et al., 2022): the inherent impossibility of definitive single-agent causal attribution given multiple concurrent seizure precipitants; (Deng et al., 2023); the absence of EEG data, precluding formal electrographic characterisation of seizure type; (Gueta et al., 2024); the valproate TDM specimen was collected at neither a true pharmacokinetic trough nor peak, constraining formal pharmacokinetic interpretation; (Hegazy et al., 2023); the incidentally detected possible left lacunar infarct on CT cannot be entirely excluded as a minor contributing factor, although the bilateral generalised semiology argues against a focal structural aetiology; (Kim et al., 2014); serum diprophylline concentrations were not measured, leaving the proposed renal impairment-driven accumulation mechanism inferential rather than directly demonstrated; and (Tassopoulou et al., 2026) although outpatient follow-up is ongoing and has thus far shown no seizure recurrence with sustained biochemical normalisation, the duration of systematic follow-up remains limited and no formal, instrument-based assessment of quality of life or psychosocial functioning was undertaken.

## Conclusion

This case suggests that renal impairment may amplify the pro-convulsant potential of diprophylline through accumulation-driven pharmacokinetic–pharmacodynamic synergy, a biologically plausible but as-yet-unconfirmed mechanism that warrants heightened clinical vigilance and renal function-guided dose adjustment whenever this agent is prescribed to patients with CKD. It further establishes that the sequential management of drug-provoked seizures and their ASM-associated complications demands a systematic pharmacovigilance framework, structured multi-confounder causality assessment at each clinical juncture, and TDM-guided individualised therapeutic decision-making. The clinical pharmacist serves as the central integrative practitioner within this process, providing the pharmacological expertise and continuous vigilance necessary to ensure safe, effective, and patient-individualised management in pharmacologically complex, high-risk clinical scenarios.

## Data Availability

The datasets presented in this article are not readily available because the data that support the findings of this case report are not publicly available due to patient privacy and confidentiality requirements. All patient data were fully de-identified prior to manuscript preparation. Further data may be available from the corresponding author upon reasonable request and subject to appropriate ethical and institutional approval. Requests to access the datasets should be directed to 20204047@zcmu.edu.cn.
